# Automatic Pulp and Teeth Three-Dimensional Modeling of Single and Multi-Rooted Teeth Based on Cone-Beam Computed Tomography Imaging: A Promising Approach With Clinical and Therapeutic Outcomes

**DOI:** 10.7759/cureus.38066

**Published:** 2023-04-24

**Authors:** Philippe Harris, Louis Harris, Jérôme Harrison, Matthieu Schmittbuhl, Jacques De Guise

**Affiliations:** 1 Faculty of Dentistry, Université de Montréal, Montreal, CAN; 2 Research Centre, University of Montreal Hospital Research Centre, Montreal, CAN; 3 Imaging and Orthopedics Research Laboratory, University of Montreal Hospital Research Centre, Montreal, CAN; 4 Medical Imaging, École de Technologie Supérieure, Montreal, CAN

**Keywords:** pulpal segmentation, dental segmentation, segmentation algorithm, image processing and analysis, cbct

## Abstract

Background

Cone-beam computed tomography (CBCT) imaging offers high-quality three-dimensional (3D) acquisition with great spatial resolution, given by the use of isometric voxels, when compared with conventional computed tomography (CT). The current literature supports a median reduction of 76% (up to 85% reduction) of patients’ radiation exposure when imaged by CBCT versus CT. Clinical applications of CBCT imaging can benefit both medical and dental professions. Because these images are digital, the use of algorithms can facilitate the diagnosis of pathologies and the management of patients. There is pertinence to developing rapid and efficient segmentation of teeth from facial volumes acquired with CBCT.

Methodology

In this paper, a segmentation algorithm using heuristics based on pulp and teeth anatomy as a pre-personalized model is proposed for both single and multi-rooted teeth.

Results

A quantitative analysis was performed by comparing the results of the algorithm to a gold standard obtained from manual segmentation using the Dice index, average surface distance (ASD), and Mahalanobis distance (MHD) metrics. Qualitative analysis was also performed between the algorithm and the gold standard of 78 teeth. The Dice index average for all pulp segmentation (n = 78) was 83.82% (SD = 6.54%). ASD for all pulp segmentation (n = 78) was 0.21 mm (SD = 0.34 mm). Pulp segmentation compared with MHD averages was 0.19 mm (SD = 0.21 mm). The results of teeth segmentation metrics were similar to pulp segmentation metrics. For the total teeth (n = 78) included in this study, the Dice index average was 92% (SD = 13.10%), ASD was low at 0.19 mm (SD = 0.15 mm), and MHD was 0.11 mm (SD = 0.09 mm). Despite good quantitative results, the qualitative analysis yielded fair results due to large categories. When compared with existing automatic segmentation methods, our approach enables an effective segmentation for both pulp and teeth.

Conclusions

Our proposed algorithm for pulp and teeth segmentation yields results that are comparable to those obtained by the state-of-the-art methods in both quantitative and qualitative analysis, thus offering interesting perspectives in many clinical fields of dentistry.

## Introduction

Cone-beam computed tomography (CBCT) imaging is finding ever-greater success in radiology with ever-widening fields of applications in both medical radiology and radiation oncology. Current CBCT imaging offers isotropic voxels of 0.05 mm. Computed tomography (CT) renders a maximum in-plane spatial resolution of 0.15 mm and a through-plane spatial resolution of 0.20 mm, whereas multi-detector CT offers 0.5 mm detector elements [1]. The higher spatial resolution of the CBCT makes it an appropriate choice for the exploration of selected small anatomical structures such as the teeth [2].

The high quality of CBCT [3] acquisitions allow for diagnostic opportunities following three-dimensional (3D) modeling of dental structures. While very high resolution facilitates dental segmentation, the prospect of routinely obtaining a 3D model of dental pulp remains a real challenge, most notably because of the often-complex shape and relative morphological variability of root canals [4].

Several approaches have been proposed in the literature for dental segmentation from CBCT acquisitions. Gao et al. proposed adaptive active contour tracking algorithms for teeth segmentation in 2010 [5]. In a 2016 study, a technique was proposed allowing tooth segmentation while having teeth contact using CT images [6]. Some literature presents active contour-based segmentation with manual initiation and custom-made constraints to allow slice-by-slice contour propagation [5-7]. A main limitation of these techniques is error accumulation.

To improve segmentation quality, a group proposed the superposition of laser-acquired dental images with CT images to create a more truthful crown reconstruction, on which CT acquisitions of the roots were merged [8]. Other similar studies have used this concept [9-11]. As outlined in our previous research, limitations of using prior shape model segmentation include requiring large databases to cope with shape variability of the natural dentition [12].

In 2019, a novel method was introduced using automatic axial slice thresholds. The proposed technique allows for rapid segmentation because the characteristics map histogram is used for optimal threshold calculations [13]. Inherent limitations of the method include cases in which pulp tissue is easily mistaken for hard tissues (e.g., calcified root canal on CBCT).

In 2021, automatic segmentation of the jaws, teeth, and CBCT background information was achieved by training a mixed-scale dense convolutional neural network [14]. This group’s algorithm yielded good results (average Dice coefficient for teeth of 0.945 ± 0.021, where 1 is better and 0 is worse). Additional use of deep learning was proposed with a multitask 3D convolutional network and marker-controlled watershed transform [15]. In recent years, several groups have reported teeth segmentation from deep-learning multi-step algorithms [10,15-18].

In this study, we aim to develop rapid and efficient segmentation of teeth from the facial volume acquired using CBCT. For this purpose, an algorithm for teeth segmentation based on pulp segmentation is proposed for both single and multi-rooted teeth.

## Materials and methods

**Figure 1:**
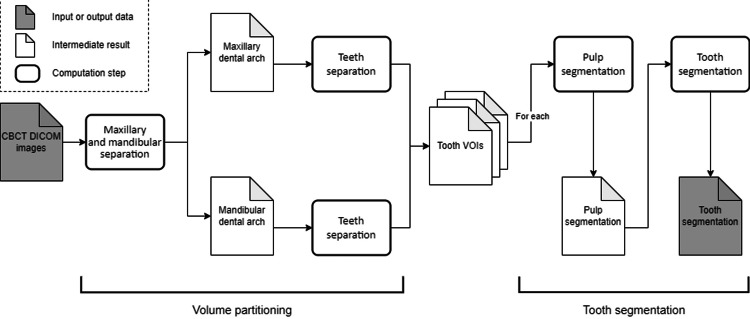
Flowchart showing a summary of the methodology.

Segmentation of both single and multi-rooted teeth takes advantage of the teeth anatomy. As for the embryologic development of teeth, we used pulps as inner structures, which have, in most cases, the same morphological outline as their corresponding teeth. As described by Harrison et al., this technique uses heuristics derived from knowledge of tooth anatomy where the pulp is used as a pre-personalized model for the tooth shape and thus helps supervise 3D segmentation [12]. The following technical description heavily relies on the proposed methodology from Harrison et al. [12]. Figure 1 summarizes the main steps of the following methodology.

Volume partitioning

Maxillary and Mandibular Separation

Separation of the dental arches (maxillary and mandible) is performed. Partitioning of the dental arches from a CBCT acquisition can be easily obtained when the patient is in an open bite position during the acquisition. In this position, maxillary and mandibular teeth do not overlap on axial slices.

Similar to the method described by Yun et al., a threshold on the maximum intensity projection (MIP) is used to differentiate between both dental arches [16]. An empirical threshold is applied on a sagittal MIP of the original Digital Imaging and Communications in Medicine (DICOM) stack. Both maxillary and mandibular dental crowns are identified with maximal intensity values. Spatial coordinates are used to train a k-nearest neighbor classifier. A dental arch shape approximation is performed by a second-order polynomial regression curve obtained from axial projection intensity levels.

Teeth Separation Planes

Once the dental arches are identified, teeth must be partitioned by finding boundary planes for adequate teeth segmentation, followed by the establishment of teeth separation planes to ensure the isolation of each tooth on the dental arch. This initial stage allows single tooth separation into a volume of interest (VOI) of its own.

As described by Kim et al. [19], candidate planes are obtained using a cost profile analysis along the second-order polynomial curves used for the shape approximation of dental arches. A Hanning filter is then used to eliminate high frequencies caused by the pulp. These planes are then rotated along the x-axis and y-axis to confirm the correct positioning of said planes. Therefore, candidate planes are described by the following equation, as presented by Harrison et al [12]:

$$C_{plane}(P_{i,\phi_1,\phi_2})=\alpha\frac{1}{N_p}\sum\limits_{n=0}^{N_p}I(\vec{p_u}) + \beta\sum\limits_{n=0}^{N_p}I(\vec{p_u})\left|\frac{\nabla I(\vec{p_u})}{|I(\vec{p_u})|}\times\vec{n}\right|$$

Where $P_(i,\phi_1,\phi_2\ )$ is a solution plane $\vec{n}$​​​​​, is the plane’s normal vector ​​​​​$\vec{p_u}$, $u\in(0,\ \ldots,\ N_p)$, is a sample point located on the plane’s surface $I\left(\vec{p_u}\right)$, and $\nabla\ I (\vec{p_u}$\)​​ are the respective grayscale and gradient value interpolated at the sample point. Weighting coefficients are applied to both terms. The cost function is evaluated for each combination of $(i,\phi_1,\phi_2)$. $C_{plane}\left({P\prime}_i\right)$ identifies the minimal cost value at every position $i$ along the central arch curve.

Each volume is then analyzed separately to perform dental segmentation, as described in the next section.

Dental segmentation

Embryology dictates that teeth are formed around the pulp. Every root and cusp has a corresponding segment of pulp. On a CBCT image, teeth appear as bright volumes and the pulp appears as a dark cavity. The method used to segment the pulp relies on this observation.

**Figure 2:**
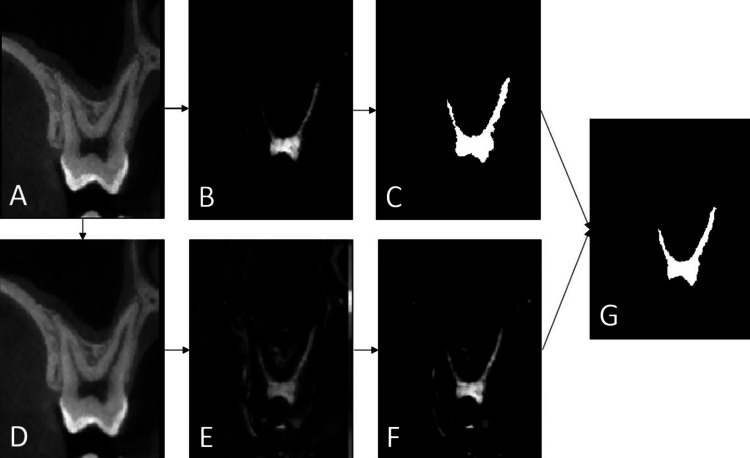
Coronal view of a molar (1.6) showing the intermediate steps involved in the pulp segmentation algorithm. (A) Original image. (B) The difference between A and hole-filled A. (C) A binary marker created from B. (D) A smoothed with a Gaussian filter. (E) D after black top hat. (F) Hole-filled E image (mask). (G) Final pulp segmentation (after masking reconstruction).

Pulp segmentation is done using morphological operations. Pulp segmentation relies on three main steps, with each performed on VOI axial slices. First, an initial marker is obtained by selecting the second-largest component in the result of the difference between the original image and the hole-filled image. Then, a mask is obtained by applying a hole-filling algorithm on top of a black top-hat transformation on the Gaussian blurred initial image. This results in an image where the valleys (dark regions enclosed in brighter ones) appear brighter. Finally, a masking reconstruction is done using the marker and the created mask. This operation consists of repeatedly performing the masking and dilatation of the marker until the result stops changing. Figure 2 shows an example of this process on tooth 1.6.

To avoid excess segmentation caused by the overflow outside of the pulp region of the hole-filled image used to create the marker, a threshold is applied to the result of the difference between the original image and the hole-filled image. The threshold value is obtained using Otsu’s method [20]. The size of the marker is also reduced to avoid overlap with an unwanted mask part used for reconstruction. Only the region around the voxel where the difference between the filled image and the original image is the highest is kept. A high difference between the filled image and the initial image implies a higher probability that this region belongs to the pulp because it is a region of low-intensity voxels surrounded by higher intensities on the original image.

Image noise and beam hardening effects can render some parts of the dentine to appear darker on images. The segmentation method considers the noise in general and includes all phenomena present in the image, with the partial volume effect being one of them. Without making any distinction between these different phenomena, the method developed nevertheless aims to, from an empirical approach of choice variations for thresholds or anatomical heuristics, optimize the quality of pulp segmentation. On the valley-enhanced image, these regions appear connected to the pulp, thus leading to a segmentation overflow. To limit this problem, a mask is applied on top of the segmentation. The mask is constructed keeping only voxels where the original image values are greater than a threshold. This value is set by taking the midpoint value between the average voxel value in regions segmented by the initial marker and the average voxel value in surrounding regions. This mask is only applied in the crown region of the tooth volume to avoid cutting root segmentation. The tooth’s orientation is obtained by comparing the first slice index corresponding to the pulp chamber, found by searching for the slice with the largest single segmented area, to the index of the first and last slices where there is a segmentation. Starting from the identified axial plane and going toward the roots, the last slice with a single segmented zone is considered the apical limit of the crown region.

**Figure 3:**
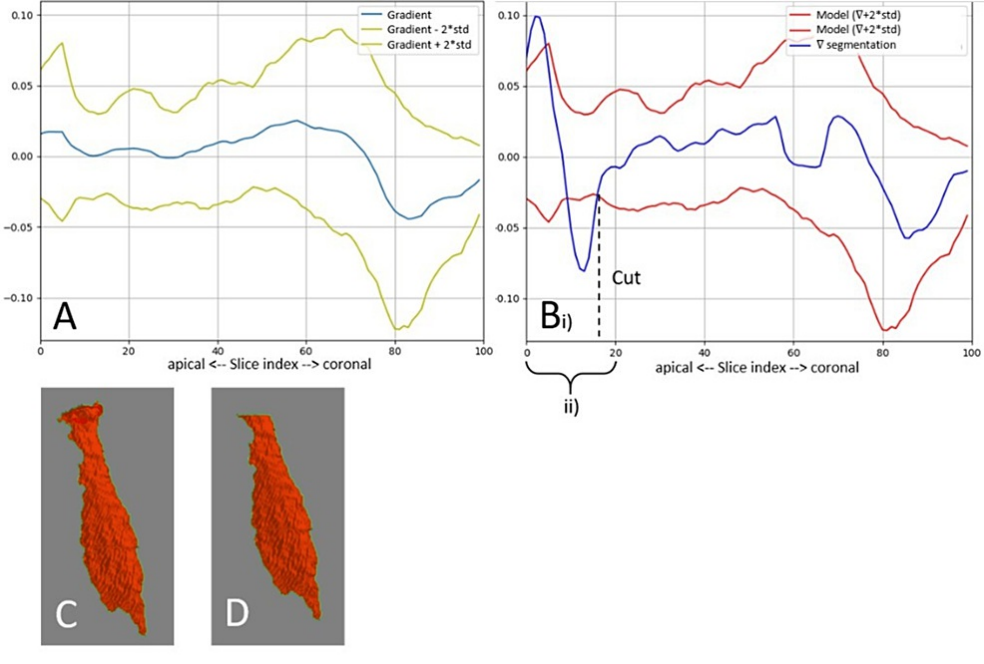
Area evolution model and its application to cut an apical segmentation overflow. (A) Model with a gradient and margin of two standard deviations. B (i): Cut of the apical overflow and (ii) analyzed region. (C) Segmentation before the cut. (D) Segmentation after the cut.

Apical overflows can occur because of the lower contrast between pulp and dentine in root canals or the opening of the apex. These overflows are characterized by a rapid change in the segmented pulp shape. To limit this problem, the obtained segmentation is compared to a slice area evolution model. This comparison is not done when the tooth is a molar because these can have very different morphologies which makes it very complex for model creation. Ground truth manual segmentations are used to create a model for each tooth type and position. For each reference segmentation corresponding to the targeted tooth type and position, the area of each axial slice of the reference mask is calculated. The resulting vectors are normalized, and the values are interpolated to obtain 100-element long vectors. The mean and standard deviation (SD) of vector gradients are computed to obtain a model of the speed of evolution of slice areas. The same gradient, normalization, and interpolation calculations are applied to the automatic pulp segmentation result. The resulting vector is smoothed with a uniform filter and is compared to the model’s vectors. Only the slices belonging to the last fifth of the mask from the root’s apex are considered for comparison. No cut is made if the obtained gradient is within two SDs of the mean of the model’s gradient. Otherwise, the cut is made at the most coronal deviation. The corresponding zone is removed from the segmentation. Figure 3 exemplifies this process.

The pulp segmentation is used as an in situ before tooth segmentation using hierarchical surface deformation [21]. The initial segmentation is iteratively deformed to obtain a final mesh representing the tooth segmentation. Each iteration consists of two main steps. First, an intensity profile search is performed to obtain a target boundary to which vertices are moved toward. This method is based on the proposed implementation presented by Chav et al. [21]. The intensity profiles $P(u,v)$ are described by the following equation: 

$$P(u,v)=\Omega(M_x,M_y,M_z)=\Omega(n_{v_{u}}\cdot u^T+v_x,n_{v_{u}}\cdot u^T+v_z)$$

Where $\Omega$ is a linear interpolation method and $u=\left[-L^{iter},\ldots,L^{iter}\right]$ is the length along which the search is performed along the normal $\vec{n_v}$ of the vertex $v_i$. The parameter $L^{iter}$ is reduced at every iteration to limit the risk of passing over the tooth’s limit. A gradient and Gaussian-based cost function is used to determine the position of a potential characteristic of interest $g_i$ along the computed profile.

To limit the over-segmentation that can occur where crowns touch their neighbors, the set of points of an intensity profile crossing the tooth separation plane is penalized. These points are ignored when performing a gradient search to prevent a vertex from being moved outside of the tooth’s VOI. Points meeting the following criteria are excluded: 

$$a_{i_{loc}}^\prime\cdot M_x+b_{i_{loc}}^\prime\cdot M_y+c_{i_{loc}}^\prime\cdot M_z-d_{i_{loc}}^\prime>0$$

Where $a_{i_{loc}}^\prime,b_{i_{loc}}^\prime,c_{i_{loc}}^\prime$ and $d_{i_{loc}}^\prime$ are constants of adjacent plane $P_{i_{loc}}^\prime$.

Evaluation of pulp and teeth segmentation

Our algorithm is evaluated quantitatively and qualitatively on a dataset of 78 teeth and compared against the manual segmentation of pulp and teeth performed by a subject matter expert having significant dental anatomy knowledge.

**Figure 4:**
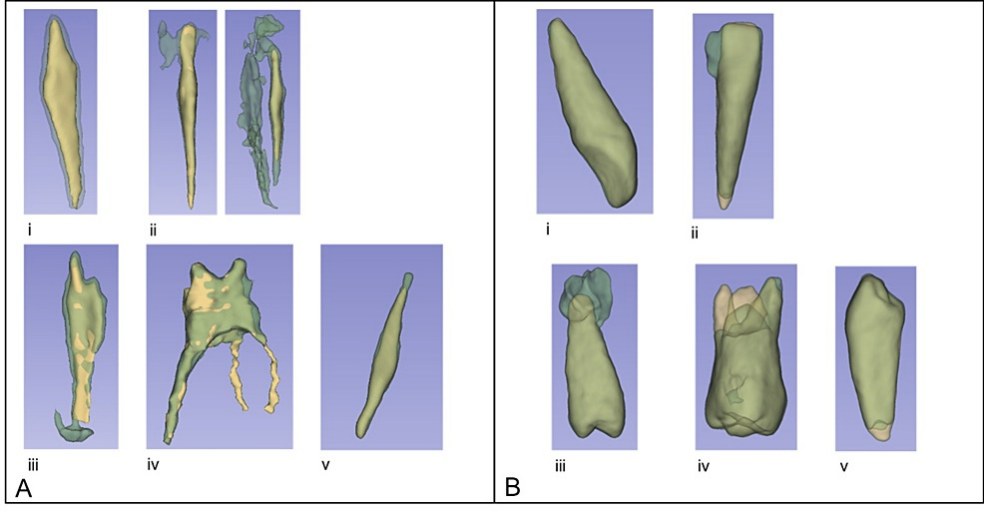
For (A) pulp segmentation and (B) tooth segmentation, examples of categories for (i) good, (ii) crown overflow, (iii) apical overflow, (iv) missing canal, and (v) missing canal extremity.

Quantitative evaluation was done using three metrics [22]. The Dice index is a value varying between 0 and 1 used to express the amount of overlap between the automatic and the reference segmentation. The average symmetric surface distance (ASD) measures the average distance between the surface of the two segmentations. The Mahalanobis distance (MHD) measures the distance between point clouds which reduces the importance accorded to local differences and gives a better indication of the global similarities between the automatic and manual segmentation results. One author (with significant dental anatomy knowledge) manually segmented pulp and teeth volumes using 3DSlicer software [23], thus rendering a reference dataset. Qualitative evaluation is done by one expert reviewer based on a subjective visual comparison of the 3D automatic segmentation results (both initial and improved algorithms) to the reference manual segmentation using the 3DSlicer software [23]. Each segmentation result is categorized into one of the eight categories described in Table 1. Examples of these categories are illustrated in Figure 4.

**Table 1:** Categories used for the qualitative evaluation of automatic segmentation results.

Category	Description
Good	The result is globally well-aligned with the reference segmentation
Apical overflow	The segmentation overflows from the root apex
Crown overflow	The segmentation overflows from the crown
Missing canal	One or more root canal is missing
Missing canal extremity	The extremity of one or more root canal is missing
Segmentation error	The automatic segmentation has failed
Other	Other segmentation errors (e.g., scattered overflows, missing parts)

## Results

The initial data included 83 teeth. However, three teeth were rejected because the volume-partitioning phase extracted the neighboring teeth. Two other teeth were rejected because the result of the pulp segmentation was outside the tooth, making the tooth segmentation impossible. These five teeth were not considered when computing quantitative and qualitative metrics for both teeth and pulp segmentation because they are considered outliers.

Quantitative analysis

Pulp Segmentation

Table 2 shows the quantitative results of pulp segmentation. The Dice index average for single-rooted teeth (n = 46) was 83.97% (SD = 7.49%). For multi-rooted teeth (n = 32), the Dice index average was 83.61% (SD = 4.98%). For total teeth (n = 78), the Dice index average was 83.82% (SD = 6.54%).

**Table 2:** Pulp segmentation metrics. SD: standard deviation; ASD: average surface distance; MHD: Mahalanobis distance

	Single-rooted (n = 46)				Multi-rooted (n = 32)			Global (n = 78)
	Incisor (n = 22)	Canine (n = 16)	Premolar (n = 8)	All types	Premolar (n = 11)	Molar (n = 21)	All types	
Dice (%) (Higher is better)								
Minimum	75.89	41.26	78.32	41.26	72.40	69.48	69.48	41.26
Maximum	90.61	89.62	88.84	90.61	85.39	89.53	89.53	90.61
Average	84.69	83.18	83.55	83.97	79.83	85.58	83.61	83.82
SD	4.45	11.54	3.54	7.49	4.60	3.98	4.98	6.54
ASD (mm) (Lower is better)								
Minimum	0.10	0.12	0.11	0.10	0.17	0.12	0.12	0.10
Maximum	0.43	3.10	0.18	3.10	0.41	0.34	0.41	3.10
Average	0.16	0.34	0.14	0.22	0.25	0.18	0.20	0.21
SD	0.07	0.74	0.02	0.44	0.08	0.05	0.07	0.34
MHD (mm) (Lower is better)								
Minimum	0.02	0.03	0.04	0.02	0.08	0.06	0.06	0.02
Maximum	0.60	1.66	0.17	1.66	0.53	0.45	0.53	1.66
Average	0.14	0.22	0.10	0.16	0.33	0.19	0.24	0.19
SD	0.12	0.39	0.04	0.25	0.14	0.10	0.13	0.21

ASD for pulp segmentation was 0.22 mm (SD = 0.44 mm) for single-rooted teeth (n = 46). Multi-rooted teeth (n = 32) yielded an average ASD of 0.20 mm (SD = 0.07 mm).

Pulp segmentation compared with MHD averages of single-rooted teeth (n = 46) yielded 0.16 mm (SD = 0.25 mm). The multi-rooted teeth (n=32) average was 0.24 mm (SD = 0.13 mm). All teeth (n = 78) MHD average was 0.19 mm (SD = 0.21 mm).

Teeth Segmentation

Teeth segmentation metrics presented similar results as pulp segmentation metrics (Table 3). The Dice average for single-rooted teeth (n = 46) was 93.75% (SD = 6.47%). For multi-rooted (n = 32) teeth, the Dice average was also high at 89.50% (SD = 18.83%). Accordingly, the total teeth (n = 78) Dice average was 92.00% (SD = 13.10%).

**Table 3:** Tooth segmentation metrics. SD: standard deviation; ASD: average surface distance; MHD: Mahalanobis distance

	Single-rooted (n = 46)				Multi-rooted (n = 32)			Global (n = 78)
	Incisor (n = 22)	Canine (n = 16)	Premolar (n = 8)	All types	Premolar (n = 11)	Molar (n = 21)	All types	
Dice (%) (Higher is better)								
Minimum	91.55	54.08	82.88	54.08	17.28	92.11	17.28	17.28
Maximum	96.34	96.77	96.12	96.77	96.06	96.68	96.68	96.77
Average	94.76	92.71	93.05	93.75	78.82	95.09	89.50	92.00
SD	1.36	10.38	5.15	6.47	30.06	1.41	18.83	13.10
ASD (mm) (Lower is better)								
Minimum	0.10	0.12	0.12	0.10	0.13	0.11	0.11	0.10
Maximum	0.20	1.32	0.54	1.32	0.53	0.26	0.53	1.32
Average	0.15	0.23	0.21	0.19	0.25	0.17	0.20	0.19
SD	0.03	0.29	0.16	0.18	0.13	0.05	0.09	0.15
MHD (mm) (Lower is better)								
Minimum	0.05	0.01	0.02	0.01	0.04	0.01	0.01	0.01
Maximum	0.23	0.44	0.54	0.54	0.20	0.21	0.21	0.54
Average	0.09	0.12	0.16	0.11	0.13	0.08	0.10	0.11
SD	0.05	0.10	0.20	0.11	0.04	0.05	0.05	0.09

Single-rooted teeth (n = 46) ASD was 0.19 mm (SD = 0.18 mm). Multi-rooted teeth (n = 32) ASD was 0.20 mm (SD = 0.09 mm). Hence, overall teeth (n = 78) ASD was low at 0.19 mm (SD = 0.15 mm).

Finally, single-rooted teeth (n = 46) MHD was 0.11 mm (SD = 0.11 mm). Multi-rooted teeth (n = 32) MHD was 0.10 mm (SD = 0.05 mm). Accordingly, total teeth (n = 78) MHD was 0.11 mm (SD = 0.09 mm).

Qualitative analysis

Segmentation results for the 78 teeth and pulps were analyzed based on the subjective quality of segmentation. The analysis classified the results as either (1) good, (2) apical overflow, (3) crown overflow, (4) missing canal, (5) missing canal extremity, (6) segmentation error, or (7) other. Analysis was performed subjectively by one expert reviewer based on a 3D rendering of segmented volumes with both initial and improved algorithms. Results yielded 39 good pulp segmentations (Table 4) and 34 good teeth segmentations (Table 5).

**Table 4:** Qualitative results of pulp segmentation.

Category	Single-rooted (n = 46)				Multi-rooted (n = 32)			Total (n = 78)
	Incisor (n = 22)	Canine (n = 16)	Premolar (n = 8)	All types	Premolar (n = 11)	Molar (n = 21)	All types	
Good	14	11	5	30	2	7	9	39
Apical overflow	1	0	1	2	0	0	0	2
Crown overflow	0	0	0	0	0	0	0	0
Missing canal	0	1	0	1	2	3	5	6
Missing canal extremity	7	4	2	13	7	10	17	30
Other	0	0	0	0	0	1	1	1

**Table 5:** Qualitative results of tooth segmentation.

Category	Single-rooted (n = 46)				Multi-rooted (n = 32)			Total (n = 78)
	Incisor (n = 22)	Canine (n = 16)	Premolar (n = 8)	All types	Premolar (n = 8)	Molar (n = 21)	All types	
Good	15	7	2	24	1	9	10	34
Apical overflow	0	3	4	7	0	0	0	7
Crown overflow	5	2	0	7	2	1	3	10
Missing canal	0	1	0	1	0	1	1	2
Missing canal extremity	1	2	2	5	6	5	11	16
Other	1	1	0	2	2	5	7	9

## Discussion

The main limitations of the presented algorithm occurred either with apical overflow or crown excessive segmentation. Apical overflow might be explained by the limited number of voxels creating the apex region and by the poor definition between apices and the surrounding periodontal ligament, as in the case of open apices. On the other hand, crown excessive segmentation might be explained by inherent pulp anatomy. Pulp coronal regions differ from their corresponding teeth coronal counterparts, especially in the posterior where each cusp has its corresponding pulp projection. Pulps are not consistent and greatly differ both between individuals and among one individual. For a given tooth, several different pulp pathways exist and might not be reflective of the corresponding teeth shapes. Therefore, teeth segmentation solely based on pulp segmentation might be biased for embryological reasons.

Overall low MHD may be suggestive of a more realistic pulp segmentation based on results provided by the expert segmentation with the optimized algorithm. A high Dice index average can be explained by high numbers of minimums which allow for high Dice index results.

**Figure 5:**
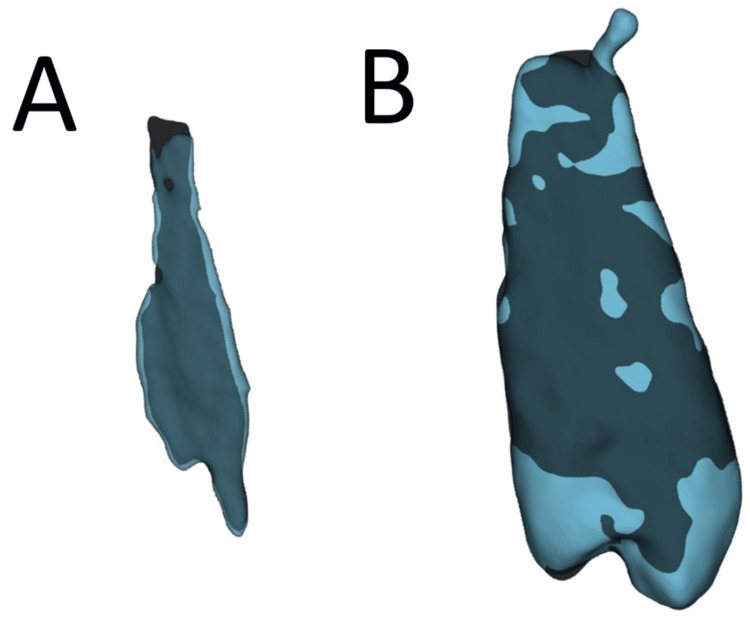
Three-dimensional rendering of segmented (A) pulp and tooth 2.4. (B) Overlapping of expert’s segmentation (dark blue) and segmentation algorithm result (light blue).

Despite good quantitative results, the qualitative analysis yielded fair results due to large categories. For example, the amount of apical overflow was not measured, and therefore, minimal over-segmentation (Figure 5A) was classified in the same category as excessive over-segmentation (Figure 5B).

When compared with existing automatic segmentation methods, as previously described, our approach enables an effective segmentation for both pulp and teeth. Despite slightly lesser precision on the Dice index (our method: 91.17% ± 14.99%; Wang et al.: 94.5% ± 2.1%) [14] compared to deep learning algorithms, our technique does not require training of a neural network, thus offering a readily exploitable automatic segmentation algorithm. Additionally, to our knowledge, no adequate CBCT image dataset is available for neural network training.

In a clinical context, pulp modeling has many diagnostic and therapeutic interests. Probably the most frequent and clinically relevant encountered variations of pulp morphology are calcified root canals or accessory canals (MB2). Variations in shape and pulp size can help clinicians in diagnostic approaches, especially in the detection of abnormalities constituting, often complex, syndromic phenotypic tables. Of note, one of the best-known pulp abnormalities is perhaps taurodontism, which corresponds to an increase in the pulp size. The detection of this pulp defect, sometimes difficult to assess because of its gradient of phenotypic expression, may be a contributing factor in the diagnosis of some syndromes in the ectodermic dysplasia group (e.g., tricho-dento-osseous syndrome, hypohidrotic ectodermic dysplasia) [24-27] or other syndromes such as Down syndrome [28], hypophosphatemia [29] and Nance-Horan syndrome [30,31]. At the therapeutic level, rapid access to pulp modeling can allow the dentist or endodontist to better appreciate the morphology of the root system, anticipate its anatomical difficulties, and, thus, facilitate treatment planning [32-35].

Other disciplinary fields can benefit from this pulp modeling and more specifically from the quantification of pulp volume. In physical anthropology, variations in pulp size can be as much a marker of sexual dimorphism as well as inter-population differences that can improve the understanding of the polymorphism of the current human species [36-40]. These markers can also have a great deal of interest in forensic science, particularly in the field of forensic identification [41-43].

Teeth 3D modeling from CBCT acquisitions would provide clinicians with additional therapeutic possibilities while improving existing procedures. A brief overview of those clinical applications includes diagnostic and planning tools or procedure aids. In an orthodontic context, current scanning technologies allow for fast and reliable data for virtual diagnosis and virtual treatment planning [44]. However, imaging of the dental arches by way of intraoral scanning devices limits the interpretation of roots’ movements in relation to adjacent anatomical structures and surrounding periodontium. Furthermore, digital scanning and CBCT of the dental arches enable proper diagnosis and construction of surgical splints [45]. Additionally, in cases such as Wilcko’s periodontally accelerated osteogenic orthodontics [46], proper root segmentation may enable advanced planning and use of surgical guides, as required. Furthermore, in case of immediate implant placement, the virtual tooth extraction based on teeth 3D modeling allows for better surgical planning, makes the procedure more accurate, and reduces surgery time.

For all these clinical and biological issues, numerical segmentation and pulp modeling are fundamental issues. A robust, fast, and accessible method to generate from CBCT images, a 3D model of teeth pulp could indeed be of great help.

## Conclusions

Our segmentation algorithm is a readily exploitable automatic segmentation tool that does not require the training of a neural network. It yields comparable results to the state-of-the-art on both qualitative and quantitative analysis. Validation against an expert’s database demonstrated the consistency and accuracy of this fully automatic teeth segmentation algorithm. Further research should include improvement in the apical segmentation of multi-rooted teeth.
